# *Cephalotaxus griffithii* Hook.f. needle extract induces cell cycle arrest, apoptosis and suppression of hTERT and hTR expression on human breast cancer cells

**DOI:** 10.1186/1472-6882-14-305

**Published:** 2014-08-18

**Authors:** Dinesh Singh Moirangthem, Surbala Laishram, Jagat Chandra Borah, Mohan Chandra Kalita, Narayan Chandra Talukdar

**Affiliations:** Institute of Bioresources and Sustainable Development, Takyelpat Institutional Area, Imphal, 795001 Manipur India; Department of Biotechnology, Gauhati University, Guwahati, 781014 Assam India

**Keywords:** *Cephalotaxus griffithii*, Apoptosis, Cell cycle, Telomerase, p53

## Abstract

**Background:**

*Cephalotaxus* spp. are known to possess anticancer potential. In this present work, for the first time the effects of *C. griffithii* needle (CGN) extracts on human cancer cells were examined.

**Methods:**

The CGN was successively extracted with petroleum ether (PE), acetone and methanol. The extracts were tested for its effect on proliferation of cancer cells (MTT assay on HeLa, ZR751 and HepG2). Extract that showed the maximum growth inhibitory effect was subjected for mechanism of action study. These included apoptosis (morphological and DNA fragmentation assay), cell cycle (flow cytometry), caspase expression (Western blot) and activity (assay kit), p53 (western blot and TP53 siRNA interference) and telomerase expression (reverse transcriptase PCR) analysis.

**Results:**

Among the extracts, PE extract induced maximum cytotoxicity, with highest death occurred in ZR751 cells. Since, PE extract induced cell death was highest among the CGN extracts, with maximum cancer cell death occurred in ZR751 cells; we carried out mechanism study of PE extract induced ZR751 cell death. It was observed that PE extract induced ZR751 cell death was associated with cell cycle arrest and apoptosis by activating both intrinsic and extrinsic apoptotic pathways. Knock down study revealed that p53 is essential for loss of ZR751 cell viability induced by PE extract. Further, PE extract down-regulated hTERT, hTR, and c-Myc expression. Thin layer chromatography analysis indicated the presence of unique phytochemicals in PE extract.

**Conclusion:**

Based on the observations, we concluded that PE extract of *C. griffithii* needle contains important phyto-components with multiple cellular targets for control of breast cancer and is worthy of future studies.

**Electronic supplementary material:**

The online version of this article (doi:10.1186/1472-6882-14-305) contains supplementary material, which is available to authorized users.

## Background

Mortality due to cancer is becoming unacceptably high and is therefore a worldwide concern. Statistics indicate that the total number of cancer deaths in 2007 was 7.6 million, of which 62% were in developing countries and 38% in developed countries [1]. By 2050, 27 million new cases and 17.5 million cancer deaths are expected globally [1]. Therefore, serious efforts have been made to reduce the threat of cancer all over the globe.

Historically, natural products have served as a rich source of lead compounds for drug development against a wide array of biological targets, including various forms of cancer. Search continues in rigorous footing to discover unexplored plants and animals as potential new sources of anticancer drugs. Since 1940, almost 75% of approved small molecules for the treatment of cancer have been either natural products, semi-synthetic derivatives of natural product scaffolds, or synthetic compounds inspired by natural products pharmacophores [2].

The genus *Cephalotaxus* has received a great level of scientific interest as it contains anticancer potential ingredients [3–6]. Homoharringtonine, an alkaloid isolated from *Cephalotaxus harringtonia* was recently approved by USFDA for the treatment of adult patient with chronic myeloid leukemia [7]. In view of the importance of the *Cephalotaxus* genus, we searched for unexplored species within this genus to check for anticancer potential components. *Cephalotaxus griffithii* Hook. f., a gymnosperm in the family Cephalotaxaceae, is another important species commonly known as Griffith's plum yew. It is a shrub or small tree and found up to an altitude of 2000 m and is distributed in North East India, western Sichuan province in China, and Myanmar [8]. *C. griffithii* mostly remained unexplored due to remoteness of location and limited accessibility of the habitat of this species. So far, only three studies from *C. griffithii* have been attempted. Kamil et al. [9] isolated and characterized six flavonoids, Phutdhawong et al. [10] carried out chemical analysis of volatile oil from needles of *C. griffithii.* Moirangthem et al. [11] analysed the biological activity of the bark extracts of *C. griffithii*.

Since plant constituents are distributed in different parts [12–14], needles of *C. griffithii* may also contain compounds with anticancer properties. Moreover, needles can also serve as a better source because it eliminates the risk of destruction associated with harvest of bark. In this study, we determined the effect of *Cephalotaxus griffithii* needle (CGN) extract on human cancer cells in terms of antiproliferation, cell cycle regulation, apoptosis induction and telomerase expression.

## Methods

### Chemicals

3-(4, 5-dimethyl-2-thiazolyl)-2, 5-diphenyl-tetrazolium bromide (MTT); acridine orange (AO); ethidium bromide (EB); propidium iodide (PI); and cell culture chemicals were purchased from Sigma-Aldrich Chemicals Pvt. Ltd. (Mumbai, India). Curcumin was purchased from HiMedia Laboratories Pvt. Ltd. (Mumbai, India). Proteinase-K and RNase were purchased from Bangalore Genei (Bangalore, India), and the rest of the chemicals and solvents used were of analytical grade.

### Plant material

The CGNs were collected from Kangchup Hills, Manipur, India (N24°52′10′ E093°46′12′) at an elevation of 1534.66 m above sea level. The specimen was identified by Dr. Biseshwori Thongam, Plant Systematics and Conservation Laboratory, Medicinal, Aromatic and Horticultural Plant Resources Division, Institute of Bioresources and Sustainable Development (IBSD), Manipur, India and by Dr. S.K. Verma, National Bureau of Plant Genetics Resources, Meghalaya, India. A voucher specimen (IBSD/C/102) has been deposited to the IBSD herbarium.

### Preparation of CGN extracts

The CGNs were air dried at room temperature and powdered. The powdered needles were then exhaustively extracted successively by soaking (which prevents the loss of biological activity of some heat-sensitive ingredients) in petroleum ether (PE), acetone (ACE), and methanol (MeOH) in order to fractionate the phytochemical constituents. Filtration was performed and the filtrates were concentrated *in vacuo* using a vacuum rotary evaporator (EYELA, Japan) and finally freeze dried (Thermo, Modulyod). The dried extracts were kept at 4°C until further analysis.

### Test sample preparation

Solutions of the test samples for the entire study were prepared in DMSO, except for the PE extract in which the sample was prepared in 1, 4-dioxan.

### Cell culture

The experimental cell lines were procured from the National Centre for Cell Science (Pune, India). HeLa and ZR751 cells were grown in DMEM (Dulbecco’s modified Eagle’s medium) and HepG2 was grown in RPMI-1640 medium supplemented with 10% (v/v) heat-inactivated fetal bovine serum (FBS) and 1% antibiotic antimycotic solution (10,000 U/ml penicillin, 10 mg/ml streptomycin sulfate, and 25 μg/ml amphotericin-B), and maintained at 37°C in a humidified atmosphere with 5% CO_2_/95% air.

### MTT reduction assay

Cytotoxicity analysis was determined using the MTT assay as reported by Mosmann [15]. Cancer cells grown in T-25 culture flasks were harvested by trypsinization, plated at an approximate density of 2 × 10^4^ cells/well in 96-well culture plates, and incubated for 24 h. Next the medium from each well was removed and the cells were washed twice with Dulbecco’s phosphate buffered saline (PBS). The cells were then exposed to increasing concentrations of extract (5–160 μg/ml) for 24 h. After incubation, the contents were replaced with MTT dissolved in serum-free medium (1.2 mM) after which the plates were further incubated for 3 h. The contents were then replaced with equal amounts of DMSO to solubilise the formazan grains formed by viable cells. Finally, the absorbance was read at 570 nm using a multi-well plate reader (Thermo, Multiskan spectrum). The viability percentage was calculated by using the formula below:


### Fluorescence microscopy

ZR751 cells (2 × 10^4^ cells/well) were cultured in 96-well culture plates, treated with or without test samples and incubated for 24 h. Staining was done using DNA-intercalate fluorescent dyes EB and AO [16], and analyzed under a fluorescence microscope (Nikon, TS 100-F).

### DNA fragmentation assay

For laddering experiments, ZR751 cells (2 × 10^5^ cells/well) were cultured in 6-well culture plates, treated with or without test samples and incubated for 24 h. Cells were then harvested, washed with ice-cold PBS (pH 7.2), and centrifuged at 500 g for 6 min at 4°C. The resulting cell pellet was dispersed in 30 μl of lysis buffer (10 mM EDTA; 50 mM Tris HCl, pH 7.8; 1% SDS) by gentle vortexing. 4 μl of proteinase-K (10 μg/μl) was then added to the above mixture, followed by incubation at 37°C for 1 h. Then, 2 μl of RNase (10 μg/μl) was added to the cell lysates, which were further incubated for 1 h at 57°C. After incubation cell lysates were mixed with 4 μl of 6X DNA loading dye and subjected to run at 2% agarose gel electrophoresis. The gel was then stained with ethidium bromide (0.5 μg/ml) and visualized under a gel documentation system (Bio-Rad).

### Flow cytometry

Cell cycle analysis was carried out using PI staining [17]. ZR751 cells (2 × 10^5^ cells/well) were cultured in 6-well culture plates, treated with or without test samples for 24 h. After incubation, cells were harvested and fixed in ice-cold 70% ethanol overnight at −20°C. Fixed cells were then treated with 0.5 ml of DNA extraction buffer (192 ml of 0.2 M Na_2_HPO_4_ with 8 ml of 0.1% Triton X-100 (v/v)) for 5 min at room temperature. DNA was stained with PI (0.02 mM) and incubated for 1 h in the dark. Flow cytometric analysis was then performed using a flow cytometer (BD FACSCaliber).

### Assessment of caspase activities

Caspase-3/7, caspase-8 and caspase-9 activity was assayed by measuring the light intensity using an assay kit (Caspase-Glo® 3/7Assay, Caspase-Glo® 8 Assay and Caspase-Glo® 9 Assay, Promega) with some modification. Briefly, ZR751 cells (1 × 10^5^ cells/well) were treated with different concentrations (5–40 μg/ml) of test samples for 24 h. After incubation, cell pellets were lysed in lysis buffer (50 mM Tris–HCl pH 7.4, 150 mM NaCl, 1% NP-40, 0.1% SDS, 2 mM EDTA, 1 mM phenylmethylsulfonylfluoride and protease inhibitor cocktail). Equal amount of protein extract cell lysates were loaded in opaque white 96-well plates and 40 μl of caspase-3/7 or caspase-8 or caspase-9 reaction buffer was added and incubated at room temperature for 1 h before measurement.

### RNA extraction and reverse transcriptase PCR

The ZR751 cells (2 × 10^5^ cells/well) were cultured in 6-well culture plates, treated with or without test samples for 24 h. After incubation, total RNA was prepared using an RNeasy kit (Qiagen) and primed with random hexamers to synthesize complementary DNA using superscript II reverse transcriptase (Invitrogen) according to the manufacturer’s instructions. Polymerase chain reaction (PCR) was carried out in a Mastercycler (Biorad) with specific primers (Additional file 1: Table S1). Amplification products obtained by PCR were electrophoretically separated on 1.5% agarose gel, stained with ethidium bromide (0.5 μg/ml) and visualized under gel documentation system (Bio-Rad).

### Western blot analysis

Protein expression was carried out as per the standard protocol [18]. Briefly, ZR751 cells (2 × 10^5^ cells/well) were cultured in 6-well culture plates, treated with different concentrations (5–40 μg/ml) of PE extract for 24 h. Cell pellets were lysed in lysis buffer (50 mM Tris–HCl pH 7.4, 150 mM NaCl, 1% NP-40, 0.1% SDS, 2 mM EDTA, 1 mM phenylmethylsulfonylfluoride and protease inhibitor cocktail). Equal amount of protein extract cell lysates were electrophoresed in SDS polyacrylamide gel and then transferred onto PVDF membranes. The membranes were subsequently incubated with primary antibodies of anti-p53, anti-BAX, anti-Bcl2, anti-caspase-3, anti-PARP, anti-cleaved PARP or anti-β-actin at room temperature for 60 min. Antibody recognition was detected with the anti-rabbit or anti-mouse IgG linked to horseradish peroxidase at room temperature for 60 min. Protein bands were visualized on Chemi Doc XRS Imaging System, Bio-Rad after treating with enhanced chemiluminescence reagent.

### siRNA interference

p53 expression was knocked down using validated silencer TP53 siRNA (Ambion). Sequences were: sense 5′-GUAAUCUCAUGGGACGGAAtt-3′ and antisense 5′- UUCCGUCCCAGUAGAUUACca-3′. For siRNA transfection experiments, ZR751 cells were plated and transfected after 24 h at ~70% confluency by using Lipofectactamine RNAiMAX transfection agent, according to the manufacturer's instructions. After 24 h of transfection, cells were exposed to different concentrations of PE extract for 24 h prior to analysis of protein expression by western blot or cell viability by MTT assay. A non-targeting control siRNA (SilencerH Negative Control-Ambion) was used as transfection control.

### Thin layer chromatography

Equal concentration (16 μg/μl) of CGN extracts were loaded on silica gel 60 TLC aluminium sheets. The plates were developed using petroleum ether: ethyl acetate (5:2). The spots were located by exposing the plates to UV light (365 nm).

### Statistical analysis

The values are presented as mean ± SD (standard deviation). Comparision between two groups were done by t–test and multiple comparisons between more than two groups were performed by one-way ANOVA supplemented with Tukey’s HSD test. Values at *P* < 0.05 were considered to indicate statistical significance.

## Results

### CGN extracts treatment induces death of human cancer cells

**Figure 1:**
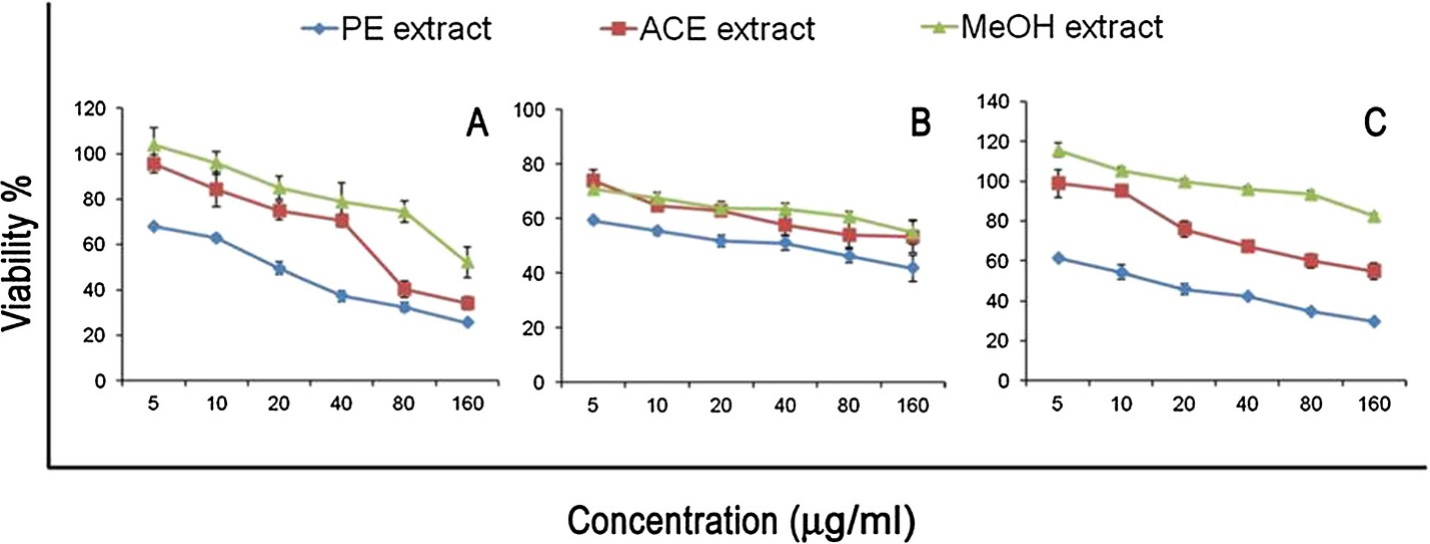
**CGN extract treatment reduces human cancer cell viability. (A)** HeLa **(B)** HepG2 and **(C)** ZR751 cells were treated with doses range from 5 – 160 μg/ml for up to 24 h prior to analysis of cell viability by MTT assay. Cell survival was determined as the percentage of the control from three independent experiments. Accordingly, IC_50_ were also determined. Each bar represents the mean ± SD (n = 3). Means marked with different letters, within each row, are significantly different (*P* < 0.05) analysed by one-way ANOVA supplemented with Tukey’s HSD test.

Effects of the three CGN extracts in six graded doses on three tumor cell lines (HeLa, HepG2 and ZR751) are shown in Figure 1. All the extracts induced death of tested cancer cells. The relative number of surviving cells decreased in a dose-dependent manner and the magnitude of effects varied depending on the cancer cells tested. Among the extracts, PE extract exhibited the maximum antiproliferative effect in all the cancer cells tested followed by ACE and MeOH extract (*P* < 0.01-0.001). PE extract induced maximum cell death in ZR751 cells (*P* < 0.001) (Figure 1; Table 1) indicating the selective cytotoxicity of PE extract towards breast cancer cells.

**Table 1:** **IC**_**50**_ **values of** ***Cephalotaxus griffithii*** **needle extracts treatment on cancer cells**

Cancer cell	Extract (μg/ml)		
	PE	ACE	MeOH
HeLa	83.5 ± 7.2p	143 ± 10.2q	205.2 ± 16.2r
HepG2	66.3 ± 6.1p	128.9 ± 13.1q	275.3 ± 19.1r
ZR751	22.3 ± 3.9p	151.4 ± 13.8q	343 ± 16.2r

Means marked with different letters, within each row, are significantly different (*P* < 0.05) analysed by one-way ANOVA supplemented with Tukey’s HSD test.

### CGN PE extract treatment induces apoptosis of human breast cancer cells

**Figure 2:**
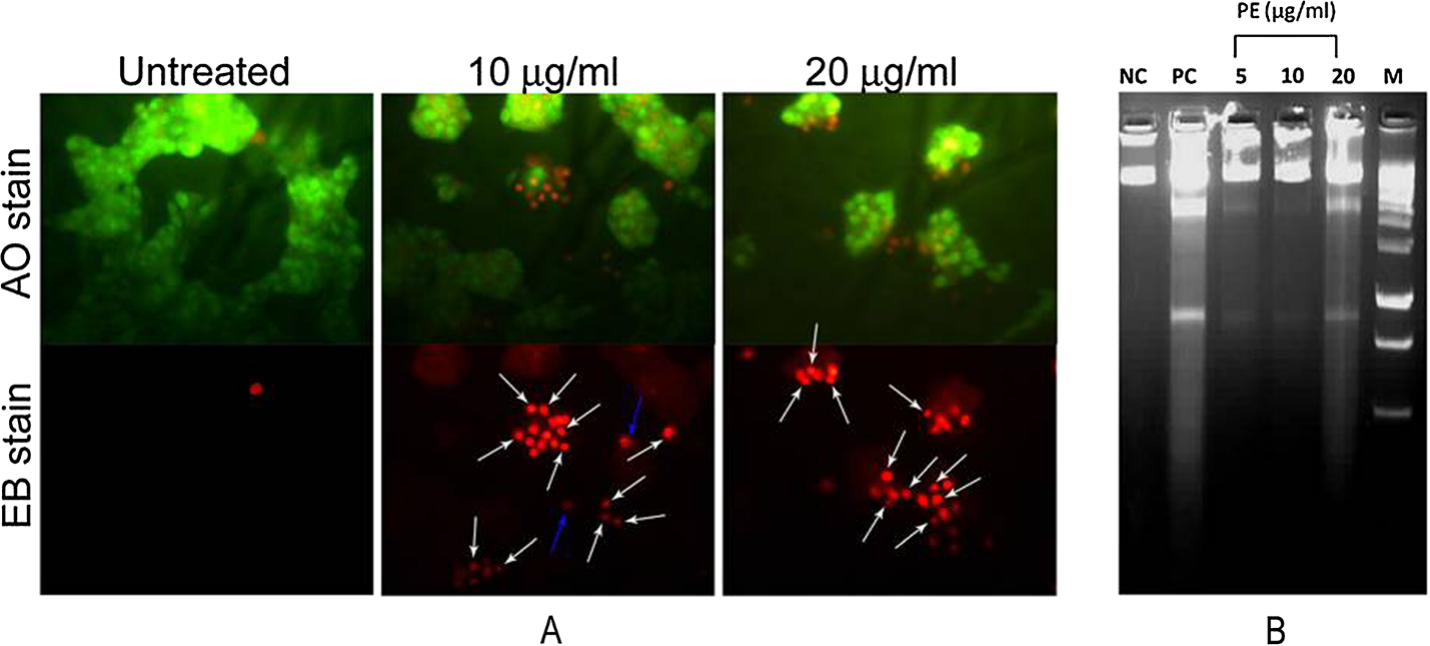
**CGN PE extract treatment induces apoptosis in human breast cancer cells. (A)** ZR751 cells were treated with (5, 10 and 20 μg/ml) or without PE extract for 24 h prior to stain with AO/EB and viewed under fluorescence microscope (400×). The treated ZR751 cells showed characteristic features of apoptosis such as nuclear condensation (white arrow) and nuclear fragmentation (blue arrow). **(B)** ZR751 cells were treated with (5, 10 and 20 μg/ml) or without PE extracts for 24 h prior to DNA fragmentation assay. The lane NC, PC, 5, 10, 20 and M represent treatment with negative control, positive control (curcumin), 5 μg/ml PE extract, 10 μg/ml PE extract, 20 μg/ml PE extract, and marker (1 kb DNA ladder), respectively.

Morphological changes of ZR751 cells caused by PE extract were analyzed using fluorescence microscope (Figure 2A) after staining the cells with AO/EB. The untreated ZR751 cells showed no contact inhibition with large nucleus. PE extract incubation led to dramatic alterations of the normal cellular architecture of ZR751 cells, and showed characteristic features of apoptosis. These included contact inhibition, nuclear contraction and nuclear fragmentation. Correspondingly, nucleosomal DNA fragmentation of ZR751 cells was also clearly visible due to the treatment with PE extract (Figure 2B).

### CGN PE extract treatment induces cell cycle arrest in human breast cancer cells

**Figure 3:**
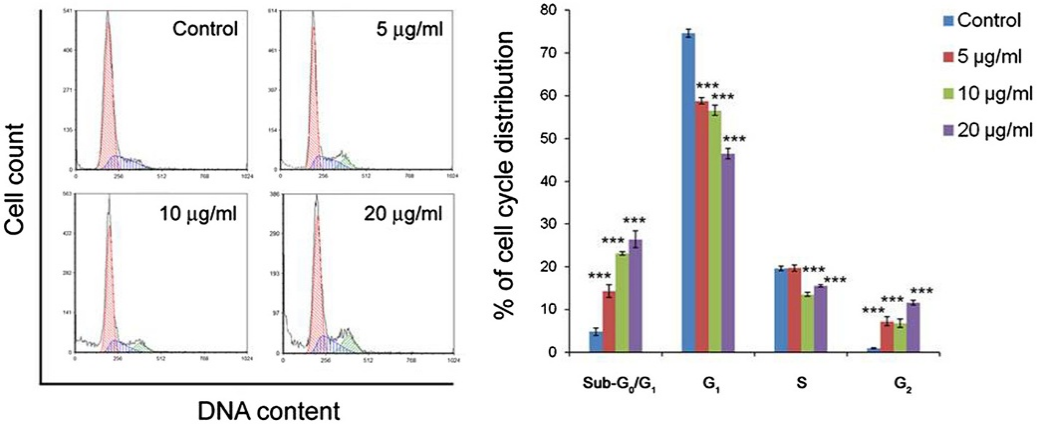
**CGN PE extract induces cell cycle arrest in human breast cancer cells.** ZR751 cells were treated with (5, 10 and 20 μg/ml) or without PE extract for 24 h prior to cell cycle analysis. Each bar represents the mean ± SD (n = 3). Data denoted ***(*P* < 0.001) are significant compared to controls, as analysed by *t*-test.

The effect of PE extract on cell cycle distribution in ZR751 cells are shown in Figure 3. Treatment of ZR751 cells with PE extract resulted in significant increase of G_2_ phase cells compared to the control (*P* < 0.001). The accumulation of cells in G_2_ increased in a dose dependent manner. This increase was coupled with the decreased percentage of cells in S and G_1_ phase (*P* < 0.001) except in 5 μg/ml treatment. Further, the sub-G_0_/G_1_ population, a biochemical marker of apoptosis [19] was significantly higher in the PE extract treated cells than the untreated control (*P* < 0.001). The increase in sub-G_0_/G_1_ population was concentration dependent. A statistically significant positive correlation was observed between the G_2_ and sub-G_0_/G_1_ cell accumulation (r = 0.905; *P* < 0.01). Overall, these results indicate that PE extract induced ZR751 cell death may be due to the induction of G_2_ cell cycle arrest and subsequent apoptotic process.

### CGN PE extract treatment activates both intrinsic and extrinsic pathways in human breast cancer cells

**Figure 4:**
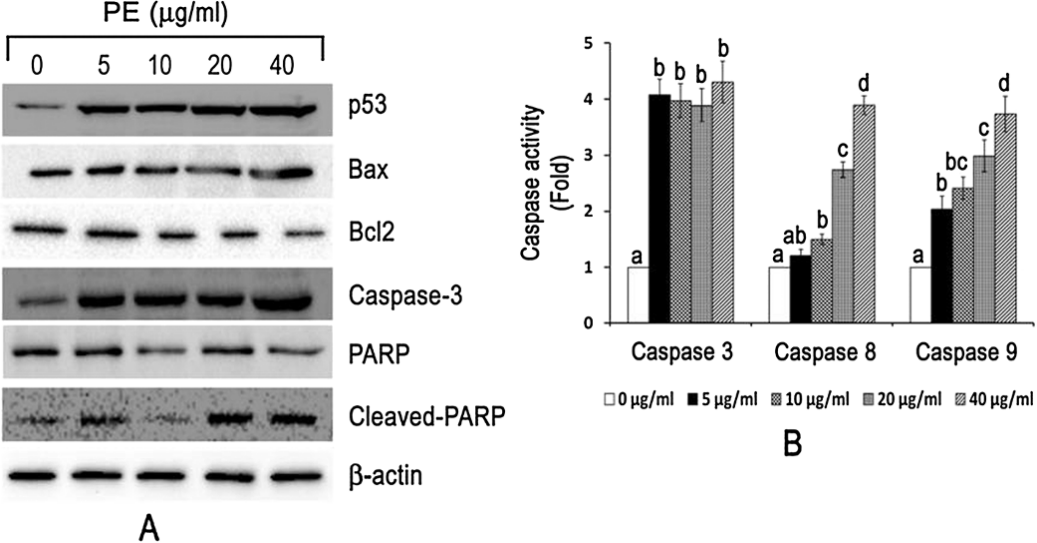
**CGN PE extract treatment causes caspase dependent apoptotic cell death in human breast cancer cells. (A)** ZR751 cells were treated with (5 – 40 μg/ml) or without PE extract for up to 24 h. Protein expression of p53, BAX, Bcl2, caspase-3, PARP and cleaved-PARP was determined by western blot. β - actin was used as loading control **(B)** ZR751 cells were treated with (5 – 40 μg/ml) or without PE extract for up to 24 h prior to caspase-3, caspase-8 and caspase-9 activity assay. Each bar represents the mean ± SD (n = 3). Bars marked without a common letter within each caspase activity are significantly different (*P* < 0.05) analysed by one-way ANOVA supplemented with Tukey’s HSD test.

Treatment with PE extract up-regulated p53 and BAX expression and down-regulated Bcl2 expression in ZR751 cells (Figure 4A). Further, incubation of ZR751 cells with PE extract resulted in the significant increase in expression level (*P <* 0.05) of caspase-3 (Figure 4A and 4B). Correspondingly, the activity of caspase-3 was also significantly increased (*P* < 0.001). A significant increase was observed even at the lowest test concentration (5 μg/ml). However, no significant dose dependent activity of caspase-3 was observed among the treated concentrations (Figure 4B). The presence of activated caspase-3 was also confirmed based on the degradation of PARP (a DNA repair enzyme), into its characteristic 89 kDa fragment, produced as a result of cleavage by caspase-3 during apoptosis (Figure 4A). Treatment of ZR751 cells with PE extract also resulted in the increased activity of caspase-8 and −9, initiator caspases, upstream activators of caspase-3/7, suggesting the involvement of both extrinsic and intrinsic apoptotic pathways. The activity of both caspase-8 and caspase-9 were increased in a dose dependent manner (Figure 4B).

### p53 is essential for loss of cell viability induced by CGN PE extract in human breast cancer cells

**Figure 5:**
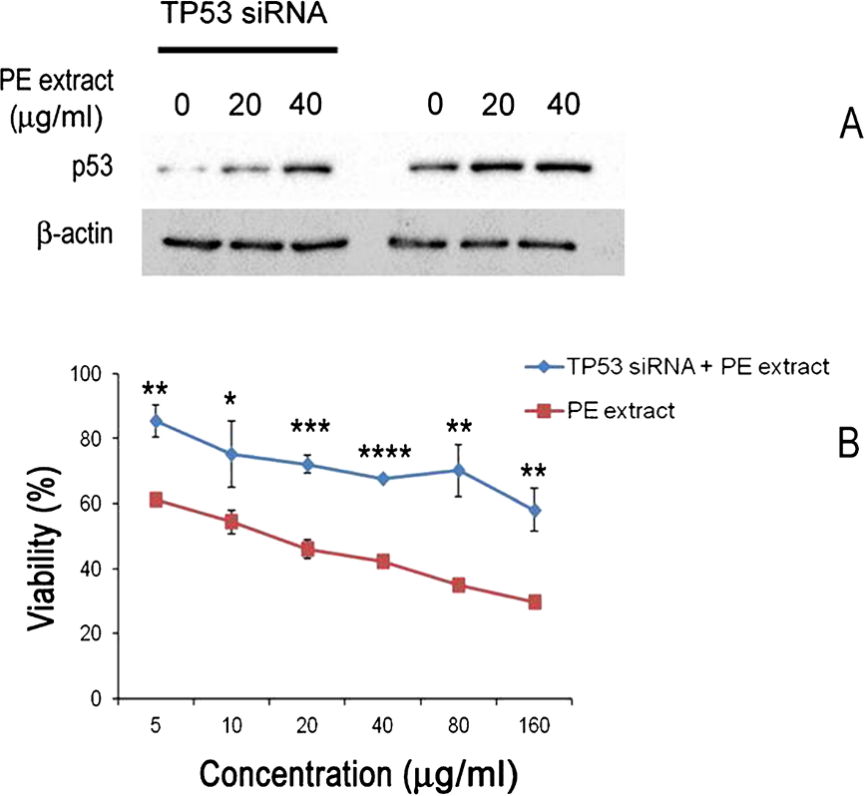
**CGN PE extract induced cellular death is dependent on p53 expression in human breast cancer cells.** ZR751 cells were transfected with TP53 siRNA for 24 h. After transfection, cells were exposed to different concentrations of PE extract for 24 h prior to analysis of **(A)** p53 expression by western blot or **(B)** cell viability by MTT assay. Each bar represents the mean ± SD (n = 3). Data denoted *(*P* < 0.05), **(*P* < 0.01), ***(*P* < 0.001) and ****(*P* < 0.0001) are significantly different compared to PE extract treatment alone (without TP53 siRNA treatment) between the same treatment concentration analysed by *t*-test.

The effect of p53 expression on antiproliferative activity of PE extract is shown in Figure 5 Silencing p53 expression resulted in significant change in the growth inhibition of PE extract on ZR751 cells. Comparing to cells with normal p53 expression, the antiproliferative activity was significantly lower in cells with silenced p53 gene (Figure 5).

### CGN PE extract treatment down-regulates hTERT, hTR and c-Myc expression in human breast cancer cells

**Figure 6:**
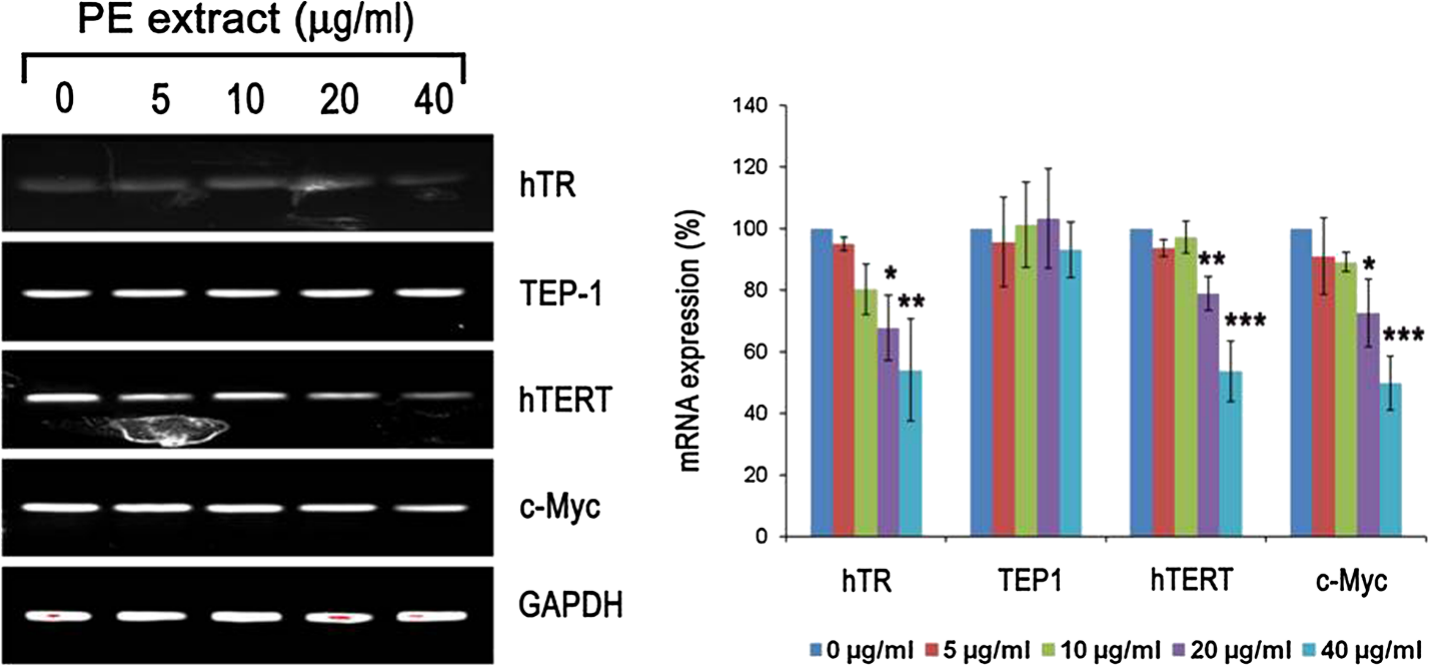
**CGN PE extract reduces hTERT, hTR and c-Myc expression in human breast cancer cells.** ZR751 cells were treated with (5 – 40 μg/ml) or without CGN PE extract for up to 24 h. mRNA expression of hTERT, TEP1, hTR and c-Myc was determined by reverse transcriptase PCR. GAPDH was used as loading control. Each bar represents the mean ± SD (n = 3). Data denoted *(*P* < 0.05), **(*P* < 0.01) and ***(*P* < 0.001) are significant compared to control analysed by one-way ANOVA supplemented with Tukey’s HSD test.

PE extract treatment decreased hTERT and hTR mRNA levels in a concentration dependent manner but had no effect on TEP-1 expressions (Figure 6) of ZR751 cells. A significant down-regulation of hTERT and hTR levels were observed due to treatment of cells at 20 μg/ml and 40 μg/ml concentrations relative to that in untreated cells. Application of PE extract also significantly down-regulated c-Myc expression in a dose-dependent manner with a significant reduction of c-Myc expression seen in the last two highest concentrations (Figure 6).

### CGN PE extract contains unique phytochemicals

**Figure 7:**
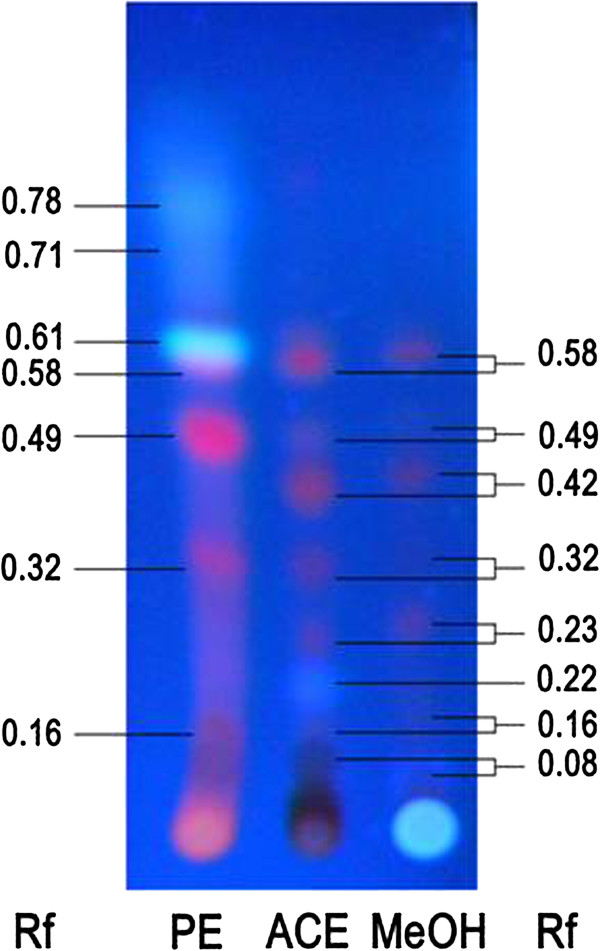
**CGN PE extract contains unique phytochemicals.** Thin layer chromatographic pattern of different CGN extracts (PE, ACE and MeOH) exposed to UV 356 nm with Rf values.

TLC profile of CGN extracts is shown in Figure 7. The chromatogram pattern of PE extract was distinctly different as compared with the other two extracts. Three spots settled at the Rf value 0.78, 0.71 and 0.61 observed in PE extract were not visible in case of ACE and MeOH extracts. There were similarities in few spots at Rf value 0.49, 0.32 and 0.16, although the intensity of the spots was much higher in PE extract.

## Discussion

This is the first report on the effect of CGN on human malignant tumor cells. For this study, the CGN was successively extracted with PE, ACE and MeOH. The antiproliferative activity was performed using MTT reduction assay on three different cancer cell lines (HeLa, HepG2 and ZR751 cells) to check the efficacy of the extracts in reducing the survival rate of the cancer cells. Irrespective of the extracts, the antiproliferative activity was found to be concentration dependent. It was also observed that the antiproliferative activity of the extracts were different and specific towards the cell type. Overall, PE extract induced the maximum cancer cell death followed by in ACE and MeOH extract. PE extract showed the highest growth inhibition towards ZR751 cells followed by HepG2 and HeLa cells. Our earlier study on *C. griffithii* bark extracts, observed that PE extract showed the least growth inhibitory effect [11] on HeLa cells. In that study, the highest antiproliferative activity was found in ACE extract treatment. This suggests that the cytotoxic components present in the needles and bark of *C. griffithii* are chemically different.

Since, PE extract induced cell death was highest among the CGN extracts, with maximum cancer cell death occurred in ZR751 cells; we carried out mechanism study of PE extract induced ZR751 cell death. Generally, anticancer agents have been reported to induce cell death by blocking cell cycle progression and triggering tumor cell apoptosis [20–22]. Therefore, cell cycle arrest and the induction of apoptosis in cancer cells become the major indicators of anticancer effects. To ascertain whether PE extract induced cytotoxicity on ZR751 cells was mediated through apoptosis and cell cycle arrest; morphological, biochemical, and cell cycle analysis were carried out. In the morphological study, most of the ZR751 cells showed characteristic features of apoptosis such as nuclear contraction, nuclear fragmentation and contact inhibition due to treatment with PE extract. Biochemically, apoptosis is characterized by activation of endogenous nucleases and fragmentation of DNA [23] which we observed in the DNA fragmentation assay on ZR751 cells after treatment with PE extract. Moreover, these fragmented nucleosomal DNA bands were very conspicuous as the concentration of PE extract increased. Further, cell cycle analysis of treated ZR751 cells with PE extract caused cell cycle arrest at G_2_ phase (*P* < 0.001). Furthermore, the sub-G_0_/G_1_ population, a biochemical marker of apoptosis [24] with hypo-diploid DNA, was significantly higher in PE extract treated cells as compared to untreated control. A positive correlation was observed between the G_2_ cell built up and sub-G_0_/G_1_ cell accumulation suggesting that the cause of ZR751 cell death by PE extract may be due to the induction of G_2_ cell cycle arrest and subsequent apoptotic process.

The p53 protein is an essential molecule that arrests cells with damaged DNA in G_2_, allowing time for DNA repair before the cell tries to enter the M phase. But if the cell is committed to division or the damage cannot be repaired, then p53 triggers a program cell death through the extrinsic and intrinsic apoptotic pathways [25, 26]. In this study, treatment of PE extract on ZR751 cells, which express a functional wild-type p53 protein [27] caused a significant increased of p53 protein expression. Further, we observed that p53 expression has a direct impact on the antiproliferative activity of ZR751 cells. When the p53 gene expression was knockdown, there was a significant reduction in the cell death induced by PE extract. This suggests that p53 is an essential target for PE extract of CGN. Among the genes activated by p53 is the pro-apoptotic protein Bax, a key member of the Bcl2 family proteins [28], and the importance of the Bax to drug-induced apoptosis is becoming increasingly apparent [29]. Bax forms a homodimer and releases cytochrome c from the mitochondria, which results in caspase-9 activation. Treatment with PE extract on ZR751 cells caused an increase in the expression of Bax which was inversely mirrored by a decrease in Bcl2 expression, an anti-apoptotic regulator. The increase in Bax expression are strongly indicative of increased p53 function whether by increased expression or activity as Bax expression can also be regulated by p53 [30].

The effector proteins in the apoptotic pathway are enzymes called caspases are a family of cysteine proteases that cleave target sites at aspartate residues, constitute key components of the apoptotic pathway. Caspase-9 triggered mitochondria pathway and the caspase-8 mediated death receptor-mediated pathway are two pathways involved in apoptosis. The caspase −9 and −8 are upstream caspases that converge to caspase-3 [26], a key effector molecule in the caspase-dependent cell apoptosis pathway that cleaves a number of cellular proteins including PARP, leading to DNA fragmentation and triggering apoptosis [31]. In this study, treatments with PE extract on ZR751 cells up-regulated the expression of caspase −8, caspase-9 and caspase-3 with the cleavage of PARP. These suggest that PE extract induced apoptosis *via* the activation of both caspase-dependent pathway through caspase-9 triggered mitochondria pathway and caspase-8 mediated death receptor pathway.

It has been reported that resistance to apoptosis is associated with telomerase expression in human cancer cells [32]. Telomeres are essential units that prevent the loss of genetic information. In normal somatic cells, which show little or no telomerase activity in synthesizing new telomeres at the ends of replicating chromosomes, the telomeric DNA progressively shortens with each cell division. Extensive shortening of telomeres is detected as a kind of DNA damage; as a result p53 is activated, leading to p53-triggered apoptosis. Most tumor cells, despite their rapid proliferation rate, stabilises by expressing telomerase [33]. It has been reported that telomerase has anti-apoptotic role and up-regulation of telomerase helps cancer cells to resist against therapeutic drug-mediated cell death. Since, telomerase is essential for a tumor cell to become immortal, and specific inhibitors of telomerase have been suggested as a new strategy for cancer therapy [34–36]. Telomere length in humans is primarily controlled by three major components, hTERT, TEP-1, and hTR. It has been shown that hTERT mRNA expression correlates with telomerase activity in human breast cancer cells [37]. In this study, we observed that application of PE extract on ZR751 cells down-regulated the expression of hTERT and hTR. These suggest that suppression of telomerase may also be one of the causes for ZR751 cell death upon treatment with PE extract of CGN. Expression of hTERT is strictly regulated at the transcriptional level by several transcription factors, particularly, c-Myc [38]. Correspondingly, with the hTERT down-regulation, the expression level of c-Myc in ZR751 cells was down-regulated after PE extract treatment.

TLC analysis revealed the presence of unique phytochemicals in the PE extract suggesting that the differences and concentration of phytochemicals in the PE extract was responsible for producing the maximum antiproliferative effect on the cancer cells.

## Conclusion

Based on our results, we concluded that CGN contain bioactive components responsible for inducing antiproliferation, cell cycle arrest, apoptosis and telomerase suppression. Among the extracts, PE extract exhibited the highest cytotoxic effect on cancer cells. PE extract induced death of ZR751 cells by acting to multiple targets *viz.* cell cycle arrest, apoptosis and suppression of telomerase. These results call for further chemical characterization of PE extract of *C. griffithii* needle for isolation of active anti-neoplastic principle.

## Electronic supplementary material

Additional file 1: Table S1: Details of the primers used for the amplification. (DOCX 14 KB)

## Abbreviations

ACE: Acetone
AO: Acridine orange
BAX: Bcl-2-like protein
Bcl-2: B-cell lymphoma 2
CGN: *C. griffithii* needle
EB: Ethidium bromide
FBS: Fetal bovine serum
hRT: Human telomerase RNA component
hTERT: Human telomerase reverse transcriptase
MeOH: Methanol
MTT: 3-(4, 5-dimethyl-2-thiazolyl)-2, 5-diphenyl-tetrazolium bromide
PARP: Poly (ADP-ribose) Polymerase
PE: Petroleum ether
PI: Propidium iodide
TEP-1: Telomerase-Associated Protein
USFDA: United States Food and Drug Administration.
