# Supplementary material for: Cephalotaxus griffithii Hook.f. needle extract induces cell cycle arrest, apoptosis and suppression of hTERT and hTR expression on human breast cancer cells
Source: BMC Complement Altern Med. 2014 Aug 18;14:305. doi: 10.1186/1472-6882-14-305 (PMC4155093; doi:10.1186/1472-6882-14-305)
Supplement: Supplementary file 1 — Additional file 1: Table S1: Details of the primers used for the amplification. (DOCX 14 KB) [file 12906_2014_1889_MOESM1_ESM.docx]

**Additional file**

***Cephalotaxus griffithii* Hook.f. needle extract induces cell cycle arrest, apoptosis and suppression of hTERT and hTR expression on human breast cancer cells**

Dinesh Singh Moirangthem ^1*^*,* Surbala Laishram ^1*^, Jagat Chaandra Borah^1^, Mohan Chandra Kalita ^2^, Narayan Chandra Talukdar ^1§^

^1^ Institute of Bioresources and Sustainable Development, Department of Biotechnology, Government of India, Takyelpat Institutional Area, Imphal, 795001, Manipur, India.

^2^ Department of Biotechnology, Gauhati University, Guwahati, 781014, Assam

*These authors contributed equally to this work

^§^Corresponding author

E-mail addresses:

DSM: [kamcha_m@yahoo.co.in](mailto:kamcha_m@yahoo.co.in)

SL: [sur_167@yahoo.com](mailto:sur_167@yahoo.com)

JCB: [jcborah03@yahoo.com](mailto:jcborah03@yahoo.com)

MCK: mckalita@sify.com

NCT: [nctalukdar@yahoo.com](mailto:nctalukdar@yahoo.com)

**Table S1** Details of the primers used for the amplification

| Gene | Sequence | Annealing temperature ( ͦ C) |
| --- | --- | --- |
| hTR | 5′-TCT-AAC-CCT-AAC-TGA-GAA-GGG-CGT-AG-3′  5′-GTT-TGC-TCT-AGA-ATG-AAC-GGT-GGA-AG-3′ | 55 |
| TEP-1 | 5′-TCA-AGC-CAA-ACC-TGA-ATC-TGA-G-3′  5′-CCC-CGA-GTG-AAT-CTT-TCT-ACG-C-3′ | 58 |
| hTERT | 5′-AGC-CAG-TCT-CAC-CTT-CAA-CC-3′  5′-GTT-CTT-CCA-AAC-TTG-CTG-ATG-3′ | 55 |
| c-Myc | 5′-AAG-ACT-CCA-GCG-CCT-TCT-CTC-3′  5′-GTT-TTC-CAA-CTC-CGG-GAT-CTG-3′ | 58 |
| GAPDH | 5′-CGG-AGT-CAA-CGG-ATT-TGG-TCG-TAT-3′  5′-AGC-CTT-CTC-CAT-GGT-GGT-GAA-GAC-3′ | 58 |
